# An Evolutionary Method for Financial Forecasting in Microscopic High-Speed Trading Environment

**DOI:** 10.1155/2017/9580815

**Published:** 2017-02-20

**Authors:** Chien-Feng Huang, Hsu-Chih Li

**Affiliations:** Dept. of Computer Science and Information Engineering, National University of Kaohsiung, Kaohsiung, Taiwan

## Abstract

The advancement of information technology in financial applications nowadays have led to fast market-driven events that prompt flash decision-making and actions issued by computer algorithms. As a result, today's markets experience intense activity in the highly dynamic environment where trading systems respond to others at a much faster pace than before. This new breed of technology involves the implementation of high-speed trading strategies which generate significant portion of activity in the financial markets and present researchers with a wealth of information not available in traditional low-speed trading environments. In this study, we aim at developing feasible computational intelligence methodologies, particularly genetic algorithms (GA), to shed light on high-speed trading research using price data of stocks on the microscopic level. Our empirical results show that the proposed GA-based system is able to improve the accuracy of the prediction significantly for price movement, and we expect this GA-based methodology to advance the current state of research for high-speed trading and other relevant financial applications.

## 1. Introduction

The advances of information technology and big data research in finance have led to an ever increasing pace to market-driven events and information that prompt decision-making and actions by computerized high-speed trading strategies. Speed has become more important to traders in financial markets because faster trading may bring about more profit opportunities, which appears to drive an arms race among traders to utilize high-speed trading technology for an edge over others. The net result is that today's markets experience intense activity in the highly dynamic environment of seconds or only a little fraction of seconds, where trading algorithms respond to others at a pace much faster than it would take for human traders to blink. This new breed of trading technology and platform involves the implementation of low-latency, high-speed trading strategies and has now resulted in remarkable portion of activities in the financial markets [1].

The time series data in high-speed trading ranges from the granular data of stock transactions at regular intervals of several seconds to the price data irregularly spaced with quotes arriving randomly at intervals of a fraction of seconds, which is mostly referred to as high-frequency trading (HFT). Over the past years, high-speed/frequency trading has been playing an important role in global financial markets due to the massive increases recently in trading volumes by such strategies. HFT has accounted for about 40% of all equity trades in the European market in 2009 [2]. As of 2013, it is reported that HFT activities accounted for 49% trading volume in the American equity markets [3]. More recently, the technologies of HFT have also been diffusing into the financial markets worldwide, including Asia [3].

In high-speed trading research, the process of price formation in market microstructure generally produces large amount of data in relatively short periods of time. The sheer volume of trading data generated in such environments provides plenty of resources for modeling and decision-making in big data research for financial applications. Recent microstructure research and advances in econometric modeling have facilitated an understanding of the characteristics of high-speed data [4]. In Taiwan, the Taiwan Stock Exchange (TWSE) is the major platform for stock trading in which transactions of various stocks typically occur at regular intervals of five seconds. In this study, we thus aim to develop novel methodology to shed light on the research in the context of high-speed trading.

In the past decades, there have been a number of computational intelligence (CI) approaches studied for financial applications due to its significant impact on the human society, ranging from fuzzy systems, artificial neural networks (ANNs), support vector machines (SVMs), and evolutionary algorithms (EAs) [5] to hybrid and ensemble models, along with other approaches [6]. These studies encompass a wide range of applications, including abnormal noise and fraud detection [7, 8], arbitrage [9, 10], bankruptcy detection [11, 12], financial forecasting [13–15], and portfolio optimization [16–18].

Although there exist many aforementioned CI methods developed for solving various financial problems, a recent survey by Aguilar-Rivera et al. [19] indicated that genetic algorithms (GA, a branch of evolutionary algorithms) have remained one of the most popular approaches in the CI literature for financial research and applications. Among several major financial areas for CI studies, forecasting is a subject that has been extensively investigated. Typically, it consists of the estimation of future values or trends of investment vehicles for relevant decision-making and investment action. Although perfect prediction is not possible, several GA-based methods have been developed to improve the accuracy of prediction. In these works, the GA techniques have been employed mainly for the optimization task in the proposed models. For instance, Kim and Han [20] proposed a GA approach to the discretization of continuous variables and the determination of optimal range for the connection weights of the ANNs to predict the stock price index. They suggested that their approach was able to reduce the numbers of attributes and the performance of forecasting was improved. In [14], Araújo and Ferreira proposed the GAs to search for optimal linear filters in forecasting applications. By comparing against several other models, they showed that their proposed GA-based models outperformed the benchmarks.

In addition to the GA-based methods, the class of Genetic Programming (GP) has been used for similar forecasting tasks, as well. For example, Shao et al. [21] proposed an improved GP-based financial forecasting tool for EDDIE (Evolutionary Dynamic Data Investment Evaluator [22]), which provided a new version of grammar to increase the search space for decision trees. In this work, a guided local search along with hill climbing was also employed to assist EDDIE with the optimization task for the rules and the forecasting time horizons. The proposed method was then tested against several financial time series and it was reported that the method was able to improve the previous version of the EDDIE for financial forecasting.

Recently, the methods of estimation of distribution algorithms (EDAs) have been studied in the area of evolutionary computation for several research problems. For financial prediction, for instance, Peralta Donate and Cortez [23] developed a NN-based forecasting approach in which a univariate marginal distribution algorithm (UMDA) was proposed for the optimization task. In this method, the best half of the current population is selected to form a portion of the new population whereas the remaining individuals are generated by the probability distribution computed by the method. Using the time series data of the Dow Jones Industrial Average, the authors compared their method with the ARIMA models, random forest, echo state networks, and SVMs and showed that their method was able to attain lower mean-squared error than others.

In addition to the EA studies discussed thus far, various types of GA-based methodologies have been developed for financial research and applications, and an extended survey is provided in [19]. However, since the advanced IT technology for fast trading platforms has been made available to public just recently, high-speed trading is still a relatively new subject to CI researchers. In particular, to the best of our knowledge, the existing major CI research has provided forecasting techniques based on the information extracted from regular, macroscopic prices, for example, daily price of a stock. In contrast, in the context of high-speed trading, the microscopic price structures are more important because the formation of the actual transaction price typically resulted from different auction prices on the microscopic level. Therefore, these microstructures shall provide more information than macrostructures for price forecasting. By this rationale, in this study, we thus aim to develop a CI-based methodology to tackle the forecasting task for high-speed trading. Since the review by Aguilar-Rivera et al. [19] indicates that the class of GA is one of the most popular approaches in CI literature for financial applications, our goal is thus to bring about a novel GA methodology to shed light on this relatively unexplored area for CI research. As our experimental results show later, using the microscopic price data from the call auction market, the methodology we proposed is indeed more effective than conventional approaches for forecasting in the context of high-speed trading.

To sum up, the overall proposed methodology in this study is to offer feasible models for the real-world high-speed trading applications. Our objective is to advance the current state of the research for the class of CI-based search algorithms particularly tailored for forecasting in the high-speed trading environment, in order to further our understanding of the complex characteristics in stock market and the applicability of the CI-based algorithms to such problems.

## 2. Materials and Methods

Currently, in the call auction market of Taiwan, the transaction prices of a stock, both the best five bid and ask prices, and their sizes are available to all market participants. In this work, we propose to use these publically available microscopic data to construct intelligent models for price forecasting. Before delving into the details of the methods studied, we provide the financial background for the call auction market first.

### 2.1. Trading Mechanism of the Call Auction Stock Market in Taiwan

In the Taiwan Stock Exchange (TWSE), the execution prices of stock trading during regular trading sessions are determined by the periodic call auction principles (http://www.twse.com.tw/en/products/trading_rules/mechanism01.php#2). Orders are collected over a specified period of time (the current period is five seconds per auction), which will be matched at the end of that period using the following rules:

1. Orders are first matched according to their price priority.
2. If the orders are of the same price, they will be matched according to their time priority.
3. For each call auction, an execution price is selected for the greatest number of orders to be executed.

In this auction system, right after the end of each matching period, a set of information is disclosed to the public, including the execution price and volume and the prices and volumes of both the five highest unexecuted bid quotes and the five lowest unexecuted ask quotes. As a result, the five best bid/ask prices and volumes observed by the public are for unexecuted orders in the prior call auction. The unexecuted orders, together with the new, subsequent orders from the investors will then enter into the system to participate in the next call auction. For illustration, Table 1 shows an example of bid and ask quotes prior to matching.

For this example, using the call auction rule for the price that enables the largest volume of orders to be executed, the system then determines the execution price to be 106.00, and 185 lots in total are executed at that price. The results for the remaining unexecuted bid and ask quotes are shown in Table 2.

Table 3 shows the disclosure of the five best unexecuted bid and ask quote prices/volumes after matching. Together with the disclosed execution price and volume, the unexecuted bid prices and volumes that are disclosed include NT$105.50, 10 lots; NT$104.50, 57 lots; NT$103.50, 30 lots; NT$102.50, 99 lots; NT$101.50, 22 lots. And the unexecuted ask prices and volumes that are disclosed are NT$107.00, 94 lots; NT$106.50, 25 lots; NT$106.00, 7 lots.

### 2.2. Stock Price Forecasting in the Call Auction Market

In the call auction market, the disclosed bid quotes contain the five highest prices and corresponding volumes for sale of the stock, and the disclosed ask quotes contain the five lowest prices and corresponding volumes for buying it. Intuitively, these bid and ask orders indicate the extent of demand and supply for a stock, respectively, which may be used to forecast the price movement in the future because the price tends to go up (or down) if the demand is more (or less) than the supply. In this paper, using the disclosed information, we thus intend to propose an intelligent GA-based system for the forecasting task of stock price.

In this section, we provide descriptions for several methodologies employed for this study: a rule-based forecasting method, two regression-based methods, and our proposed GA-based methods.

#### 2.2.1. Rule-Based Forecasting Models

As discussed in the previous section, at the end of each call auction, the disclosed information includes the execution price and volume and the five best unexecuted prices and volumes of bid and ask orders. According to the matching mechanism for the bid and ask orders, the new execution price at the next call shall be determined by the unexecuted orders at the current call and the other continuous influx of new orders entering into the system before the next call. Since the new coming orders are not disclosed to the public, market participants can only utilize the execution price and volume and the five best unexecuted bid and ask quotes at the current call for price prediction in the future. In order to predict the price at the next call, Hu and Chan [24] proposed the following rule to infer the imbalance in the order book.

**Rule 1 .**

“If a call reports both bid and ask and the transaction price is equal to the bid (ask), then the transaction price of the next call tends to go up (down).”

The rationale for this rule is that when the execution (transaction) price is equal to the bid, there are certain buying orders left unfulfilled; so these remaining demand orders may push up the price in the future. Conversely, if the execution (transaction) price is equal to the ask, there are some selling orders left unfulfilled and these supply orders then tend to push down the price. Therefore, Rule 1 may be used to predict the movement direction of the transaction price at the next call.

In addition to this method, we propose to study other versions of more sophisticated models for the call auction market. We describe the regression-based methods in the next subsection.

#### 2.2.2. Regression-Based Forecasting Models

*(A) Linear Regression Models.* Linear regression models have been studied in several financial applications, including the task of stock selection [25, 26] and the studies for the impact of order imbalance in the call auction market [24, 27]. Since the major component of this study is concerning the forecasting task under the call auction mechanism, which is similar to the ones in [24, 27] where the authors mainly used linear regression methods for their studies, in this work, we thus also propose to employ the linear regression models as follows.

Consider a given set *S* with *n* training instances {(*x*_1_(*t*), *y*_1_(*t*)), (*x*_2_(*t*), *y*_2_(*t*)),…, (*x*_*n*_(*t*), *y*_*n*_(*t*))} at time *t*. Each training instance *x*_*i*_(*t*) serves as the input to generate a corresponding output *y*_*i*_(*t*), for *i* = 1,…, *n*. Using *β* and *ε*(*t*) to denote the regression coefficients and the error terms, respectively, a linear regression model often takes the form

(1) $$\begin{array}{c} Y\left(\;t\;\right)=X\left(\;t\;\right)\beta +\varepsilon \left(\;t\;\right), \end{array}$$

where if *p* is the input dimension,

(2) Yt=y1ty2t⋮ynt,Xt=x11t⋯x1ptx21t⋯x2pt⋮⋱⋮xn1t⋯xnpt,β=β1⋮βp,εt=ε1tε2t⋮εnt.

In contrast to the simple rule-based forecasting method in the previous subsection, a more sophisticated model can be constructed through the linear regression model above. In a nutshell, the transaction price of the next call may hinge on the disclosed, best five ask (from the selling side) and best five bid (from the buying side) quotes which represent the supply and demand pressure of the stock, respectively. In this call auction market, here we designate each input variable to be the product of the bid (or ask) price and corresponding volume in order to model the degree of the buying (or selling) strength at each particular price level. Thus, these ten variables may be used as the inputs to the regression model and the price of the stock at the next call may serve as the output of the model. Therefore, once the regression model is constructed, it can be used to predict the future price of the stock as long as the values of the input variables are provided.

*(B) Logistic Regression Models.* The linear regression method may be used to model continuous variables such as the future stock price discussed above. However, other versions of regression models may be useful. For instance, if the goal is to predict future price to go up or down, then a binary output of the model may be more appropriate. In this case, a binary logistic model is studied here as an alternative to linear regression, which is used to estimate the probability of the binary response variable (price going up or down) based on the same set of input variables in the last subsection. The logistic regression model we use in this study works as follows.

Here, we consider again, in the previous subsection, the given set *S* with *n* training instances {(*x*_1_(*t*), *y*_1_(*t*)), (*x*_2_(*t*), *y*_2_(*t*)),…, (*x*_*n*_(*t*), *y*_*n*_(*t*))} at time *t*. The logistic regression employs a standard logistic function *f*(*x*), which can be defined as

(3) $$\begin{array}{c} P\left(\;X\;\right)=\frac{1}{1+e^{-\left(\beta_{0}+\beta_{1}x_{1}+\cdots +\beta_{n}x_{n}\right)}}. \end{array}$$

Note that *P*(*X*) is interpreted as the probability of the dependent variable equal to one of the binary outcomes. In this model, the 10 variables of best five ask and five bid quotes and the binary response variable *y* (price up or down at the next call) are used to compute the logistic regression model. Once the regression model is constructed, it can be used to predict the direction of price change in the future as long as the values of the input variables are provided. We will describe how to use the linear and logistic regression models for forecasting in Section 2.2.4.

#### 2.2.3. GA-Based Forecasting Models

Genetic algorithms [28] have been used as computational models of natural evolutionary systems and as a class of adaptive algorithms for solving optimization problems. GA operate on an evolving population of artificial agents. Each agent is comprised of a genotype (often a binary string) encoding a solution to some problem and a phenotype (the solution itself). GA regularly start with a population of randomly generated agents within which solution candidates are embedded. In each iteration, a new generation is created by applying crossover and mutation to promising candidates selected according to probabilities biased in favor of the relatively fit agents. As a result, evolution occurs by iterated stochastic variation of genotypes and selection of the best phenotypes in an environment according to how well the respective solution solves a problem. Successive generations are created in the same manner until a well-defined termination criterion is met. The core of this class of algorithms lies in the production of new genetic structures along the course of evolution, thereby providing innovations to solutions for the problem at hand. The steps of a simple GA are shown in the following.

**Step 1 .**

Randomly generate an initial population of l agents, each being an *n*-bit genotype (chromosome).

**Step 2 .**

Evaluate each agent's fitness.

**Step 3 .**

Repeat until l offspring have been created.

- Select a pair of parents for mating.
- Apply variation operators (crossover and mutation).

**Step 4 .**

Replace the current population with the new population.

**Step 5 .**

Go to Step 2 until terminating condition.

The GA-based methods have been widely employed to solve optimization problems and applications in computational finance and investment [19]. In this survey, the GA-based methods have been shown to be very useful in stock selection and portfolio optimization, as well as various types of financial prediction. Motivated by these research results, we intend to employ the GA to develop intelligent systems for price forecasting in this study through the optimization of parameters of the forecasting models that account for the demand and supply of a stock. More specifically, we propose to use the ten disclosed quotes (the best five bid and five ask prices and volumes) of a stock as the inputs to the GA-based model in order to predict the direction of future price movement. Here, we define the extent of the bid strength (BS) and ask strength (AS) of a stock at time *t* as follows:

(4) BSt=∑i5uibit;ASt=∑i5visit,

where *b*_*i*_(*t*) and *s*_*i*_(*t*) denote the product of price and volume (lots) for the *i*th bid and ask quote of the stock at time *t*, respectively, and *u*_*i*_ and *v*_*i*_ denote the corresponding weight for the *i*th bid and ask quote, respectively.

The difference between a stock's demand (buying) and supply (selling) power can then be defined as

(5) $$\begin{array}{c} x\left(\;t\;\right)=BS\left(\;t\;\right)-AS\left(\;t\;\right). \end{array}$$

In this study, a prediction rule for the price movement according to (5) can be proposed as follows: When the difference *x*(*t*) is positive (i.e., the demand is more than the supply), one predicts the price of the stock to go up in the future. Conversely, it is predicted to go down if *x*(*t*) is negative.

As an illustration, in Table 3, the disclosed unexecuted bids include 10 lots of $105.50, 57 lots of $104.50, 30 lots of NT$103.50, 99 lots of $102.50, and 22 lots of $101.50. These unexecuted bids represent the remaining demand force for the stock where the higher the bid order is, intuitively, the more likely it is to be matched with new ask orders in the future. Conversely, for the unexecuted ask orders, they remain as the supply force of the stock; and the lower an ask order, the more likely it is to be matched with new bid orders. Therefore, various levels of bid and ask quotes may have different degree (weight) of impact on determining the final transaction price at each call. In this study, we thus propose the model by (4) and (5) to calculate the force that potentially leads to the price change due to various levels of demand and supply. The result can be then used as the prediction for price movement of a stock (i.e., the prediction rule by (5) mentioned above); and the corresponding forecasting performance can be further evaluated by the precision measure in (7) discussed later in the next subsection (Section 2.2.4).

In order to determine the weight for each price level of bids and asks, we hereby propose the GA to search for optimal *u*_*i*_ and *v*_*i*_'s as follows.

**Figure 1:**
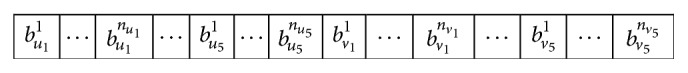
Chromosome encoding.

For the model studied here, we devise a chromosome as two portions that encode the parameters *u*_*i*_ and *v*_*i*_, *i* = 1,…, 5, for the weight corresponding to the *i*th bid and ask quotes, respectively. A binary coding scheme is used in this study to represent a chromosome in the GA. For instance, in Figure 1, loci *b*_*u*_*i*__^1^ through *b*_*u*_*i*__^*n*_*u*_*i*__^, *i* = 1,…, 5, represent the encoding configuration for *u*_*i*_; and loci *b*_*v*_*i*__^1^ through *b*_*v*_*i*__^*n*_*v*_*i*__^, *i* = 1,…, 5, represent the encoding for *v*_*i*_, where *n* represents the bit length.

In our encoding scheme here, a chromosome representing the genotypes of parameters is to be transformed into the phenotype by the following equation for further fitness computation:

(6) $$\begin{array}{c} y={min}_{y}+\frac{d}{2^{i}-1}\times \left(\;{max}_{y}-{min}_{y}\;\right), \end{array}$$

where *y* is the corresponding phenotype for the particular parameter; min_*y*_ and max_*y*_ are the minimum and maximum values of the parameter; *d* is the corresponding decimal value (*d* being truncated to integers if the parameter is of integer type), and *l* is the length of the block used to encode the parameter in the chromosome.

Once the GA is employed to search for optimal *u*_*i*_'s and *v*_*i*_'s for any prespecified objective, these GA-based models can be used for the prediction of a stock price in the future. For instance, as the new best five bid and ask quotes of a stock at the current call are made available to the public, the proposed GA-based model can use this information to calculate the difference between the demand and supply and further predict whether the price would go up or down in the future.

#### 2.2.4. Performance Measures and Forecasting Systems for Comparison

In this work, the performance of the forecasting system can be measured by the precision defined as

(7) $$\begin{array}{c} precision=\frac{TP}{TP+FP}, \end{array}$$

where TP and FP denote the number of true positives and false positives, respectively. In this study, we propose to calculate true and false positives as follows:

  “When a system predicts the price of a stock to go up at some point in the future, and if the price indeed goes up then, a true positive occurs; otherwise, it is a false positive.”

Alternatively, the performance of a system may also be evaluated by the accuracy metric defined as follows:

(8) $$\begin{array}{c} accuracy=\frac{TP+TN}{TP+TN+FP+FN}, \end{array}$$

where TN and FN denote the number of true negatives and false negatives, respectively, which can be defined as follows:

  “When the stock price is predicted to go down at some point in the future, and if the price indeed goes down then, a true negative occurs; otherwise, it is a false negative.”

In this study, we are more interested in the task of predicting the price to go up, so that one may make profit if a proper buying strategy is employed using this prediction result. Therefore, the major experimental results we present later in the Results (Section 3) center around the precision performance evaluated by (7). However, in the final portion of the Results, we also present some results using the accuracy metric by (8) for additional comparison of different systems, which is used to show that our proposed system is effective under both the precision and accuracy measures.

In addition, it is worthwhile to provide further discussions concerning some pitfalls in the high-speed trading context of the call auction market. In the stock market, there are three cases for the price movement of a stock at any moment in the future.

**Case 1 .**

The new price remains the same as the current one.

**Case 2 .**

The price goes up (i.e., the new price is higher than the current one).

**Case 3 .**

The price goes down (i.e., the new price is lower than the current one).

We notice that, in current Taiwan's call auction market, a stock's transaction price tends to remain unchanged among several consecutive calls. Thus, the distribution of the transaction price of a stock is typically skewed toward Case 1. As an illustration, we consider the stock of the Taiwan Semiconductor Manufacturing Company Limited (TSMC) from Sept./22/2015 through Nov./24/2015. If the call auction system reports that a transaction price is equal to the bid, then according to Section 2.2.1's Rule 1, the next transaction price tends to go up. We then inspect the real data under this condition and observe that the corresponding transaction prices at next calls actually consist of 41585, 14982, and 41 occurrences of price going flat, up, and down between two consecutive calls, respectively. As a result, if one designates the true positive (TP) by Section 2.2.1's forecasting rule to include both the flat case (Case 1) and up case (Case 2), then the number of TPs is raised up to 56567 and the precision is 0.99, so that the forecasting task (i.e., forecasting the price to remain the same or go up) becomes trivial. However, if the flat cases are excluded when computing TP, the resultant precision drops significantly to 0.26 and the forecasting (i.e., forecasting the price to only go up) would be difficult. Therefore, once the event of price remaining unchanged between two consecutive calls is excluded, the rate of correct prediction by Section 2.2.1's rule may drop significantly. As the experimental results show later, this challenge will be substantial to several forecasting methods in the call auction market studied here. Motivated by this, we hereby intend to propose new systems in order to improve the accuracy of prediction for the movement of stock price in the high-speed call auction market.

In this study, we will conduct a comparative study on the prediction performance for the aforementioned forecasting systems as follows.

*(i) System  1 (S1).* The forecasting rule in Section 2.2.1 is used to predict the price of a stock at the next call to go up (down) if one observes that both bid and ask are reported and the transaction price is equal to the bid (ask) at the current call.

*(ii) System  2a (S2a).* The linear regression model in Section 2.2.2(A) is employed for this system. Using both the disclosed five best bid and ask quotes at the current call as the input to the regression model, the output of the model then serves as the prediction of the price at the next call.

*(iii) System  2b (S2b).* The logistic regression model in Section 2.2.2(B) is employed for this system. Again using the 10 variables of both the five bid and five ask quotes at the current call, the logistic regression model calculates the probability of the price going up or down at the next call. Here, we predict the price to go up if the probability is greater than 0.5; otherwise, the price is predicted to go down.

*(iv) System  3 (S3).* The GA-based model in Section 2.2.3 is employed for this system. The fitness function of a chromosome is defined as the precision by (7) or the accuracy by (8). Once the GA is employed to search for the optimal parameters for the models, the resultant models may be used for the prediction of price movement of a stock in the future. Therefore, both the disclosed five best bid and ask quotes at the current call are used as the input to the model. If the model detects that the demand of a stock is larger (less) than the supply, it predicts the price to go up (down) at the next call.

*(v) System  4 (S4).* This system combines S1 and S3 to predict the price in the future. The fitness function of a chromosome is defined as the precision by (7) or the accuracy by (8). Therefore, the disclosed five best bid and ask quotes at the current call and the transaction price are used as the input to the model. Intuitively, we expect the combination of the two systems to improve either S1 or S3 alone and thus refine the forecasting system. In addition, once the system detects that the demand of a stock is larger than the supply, we expect the stock's price to go up at some point in the future, rather than the next call. In this system, we thus propose to enrich the encoding of the chromosome by including a few bits to search for the optimal period for calculating the precision.

## 3. Results

In this section, we present experimental results for the five systems described in Section 2.2.4. We use 10 stocks with large market capitalization in the Taiwan Stock Exchange for illustration, among which 5 are from the semiconductor and electronics industry and the other 5 are from the financial industry. These two industries are the two major ones in Taiwan and thus consist of a large portion of commercial activities in Taiwan. Therefore, we choose these 10 stocks in order to provide more representative characteristics of Taiwan's stock market to examine our proposed methods. The datasets are made available to the public by the TWSE where the transaction prices, five best bid and ask quotes, and trading volumes are used to examine the performance of the systems.  Table 4 shows the 10 stocks used in this study. In this study, the datasets were extracted from two periods of time (each period accounts for the total of 30 trading days): (1) Sept./15/2015 through Nov./03/2015, during which time the value of the Taiwan Stock Exchange Capitalization Weighted Stock Index (TSEC weighted index) went up from 8259.99 to 8713.19, and that is a period of time the broad stock market is achieving positive gain; (2) Dec./10/2015 through Jan./21/2016, during which time the TSEC weighted index went down from 8216.17 to 7664.01, thus the broad stock market achieving negative gain. The reason we selected these two periods of time is to examine whether our proposed method would be generally effective when the broad stock market makes either gains (i.e., the market encounters favorable conditions) or losses (i.e., the market faces adverse challenges). For each trading day, the market opens from 9:00 am through 1:30 pm. The transaction data is sampled per 5 seconds. Each sample contains the information of ticker, transaction price, number of transactions, volume, the best five ask and bid quotes, etc.

**Figure 2:**
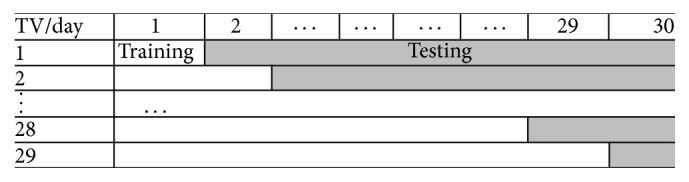
Temporal validation.

In order to examine the effectiveness of the systems studied, statistical validation is presented in this section. As shown in Figure 2, we use the data of the first several days to train the model, and the remaining data is used for testing. This setup is to provide a set of temporal validations to examine the effectiveness of the models for the dynamic characteristics in many financial applications, which is different from the regular cross-validation procedure where the process of data being split into two independent sets is randomly repeated several times without taking into account the data's temporal order [5, 10].

**Figure 3:**
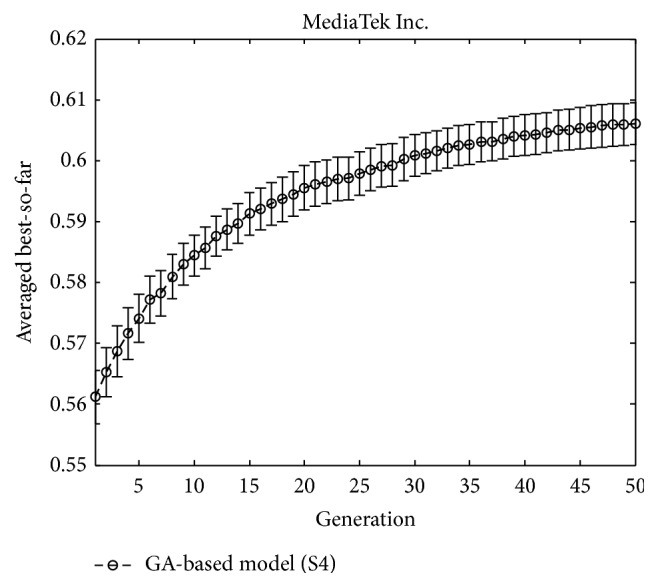
Best-so-far curve by the GA for MediaTek Inc.

In the training phase of each TV, we conduct 50 runs for the GA experiments with population size of 50 individuals in each generation. For each weighting parameter, we use 8 bits to encode it whose range is designated from 0 to 1 (using more bits are possible to offer higher resolution, but the computational overhead is increased, as well). We also use a binary tournament selection [29], one-point crossover, and mutation rates of 0.7 and 0.005, respectively (the values of crossover and mutation rates have various effects on the performance of the GA search; the two values we chose here are typical as suggested in [30, 31]). In order to track the change of the quality of solutions searched by the GA over time, a traditional performance metric for search algorithms is the “best-so-far” curve that plots the fitness of the best individual that has been seen so far by generation* n* for the GA—i.e., a point in the search space that optimizes the objective function thus far [5]. As an illustration, Figure 3 displays a typical averaged best-so-far curve over 50 runs attained by the GA in this study (these results were obtained for MediaTek Inc.; since the results for other stocks are similar, they are not displayed here). The averaged best-so-far performance curve is calculated by averaging the best-so-far solutions obtained at each generation for all 50 runs, where the vertical bars (error bars) overlaying the curve represent the 95% confidence intervals about the means. From this figure, one can clearly see the convergence of the GA search and the length of the error bars are sufficiently small, thereby indicating the GA search results are robust.

Furthermore, in this study, the best model learned in the training phase for each run is examined in the testing phase. Therefore, in both training and testing phases of each TV, the averaged fitness (precision) of the models can be calculated. For illustration, Table 5 displays the averaged precisions for Advanced Semiconductor Engineering Inc. during the period of time from Sept./15/2015 through Nov./03/2015. In this table, an inspection on the means shows that in all the 29 TVs of the training case the GA-based model S4 outperforms all the other models. For the testing phase, except TVs 19 and 23 in which S4 underperforms S2a, S4 still outperforms all the other systems in the remaining 27 TVs.

**Figure 4:**
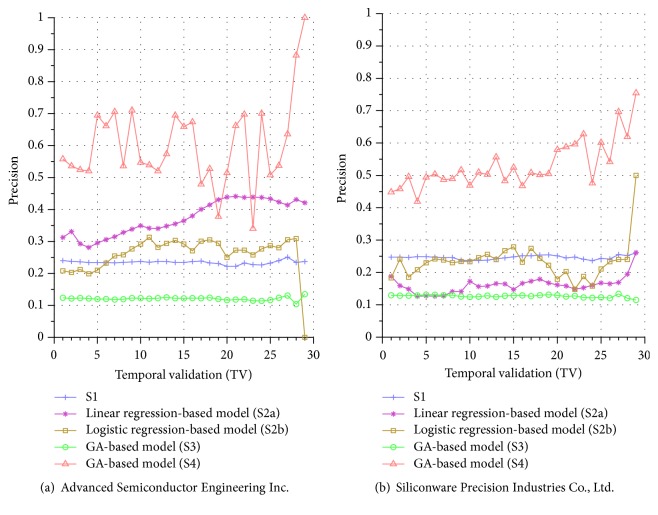
Precision comparison of the forecasting systems in the testing phase for 9/15/2015–11/3/2015.

**Figure 5:**
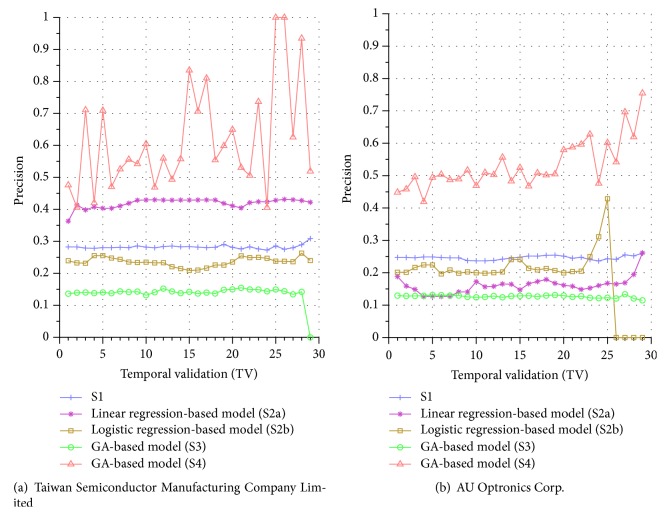
Precision comparison of the forecasting systems in the testing phase for 9/15/2015–11/3/2015.

Figure 4(a) further displays a visual gist on this performance discrepancy of the five systems in the testing phase. In this figure, we notice that all the precisions by S1 are around 0.25. For S2a, most of the precisions are between 0.27 and 0.45. For S2b, most of the precisions are between 0.2 and 0.3, except the final TV where the model did not find any TPs with testing data of only one day available. Similar phenomenon can be seen for other companies, for example, the precision of S3 in the final TV of Figure 5(a) and those of S2b in the last 3 TVs in Figure 5(b) (notice that it does not mean that a random coin-toss strategy is better than these systems; the precisions of these systems are below 0.5 because we remove the flat price cases in computing the TP, as discussed in Section 2.2.4, which thus presents substantial challenge to the forecasting task).

For S3, the results are quite poor where all the precisions are between 0.1 and 0.15. An investigation into these unsatisfactory performances indicates that even though the model detects that the demand for a stock is more than the supply and thus expects the price to go up, the price may still remain unchanged for next several calls and finally go up after that. In other words, using the price of the next call may not be the best timing for calculating precision. Therefore, in S4, our proposed system allows the GA to evolve a better timing for computing the precision and the results indeed show that it improves the performance of the system significantly. However, we also notice that the precisions suffer high variance, indicating that the performance of system S4 is not very stable across various TVs (different TVs have different number of training and testing days). This is perhaps because the period of the testing phase is not long enough for S4 to exhibit stable performance in the final few TVs, and we intend to investigate this issue in more detail in the future.

**Figure 6:**
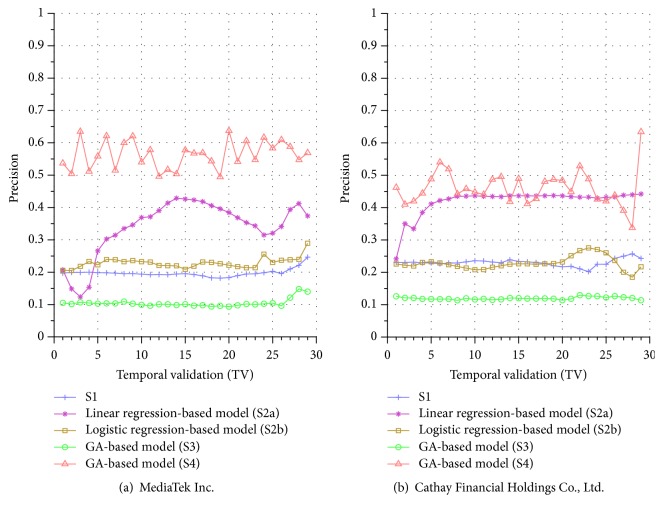
Precision comparison of the forecasting systems in the testing phase for 9/15/2015–11/3/2015.

**Figure 7:**
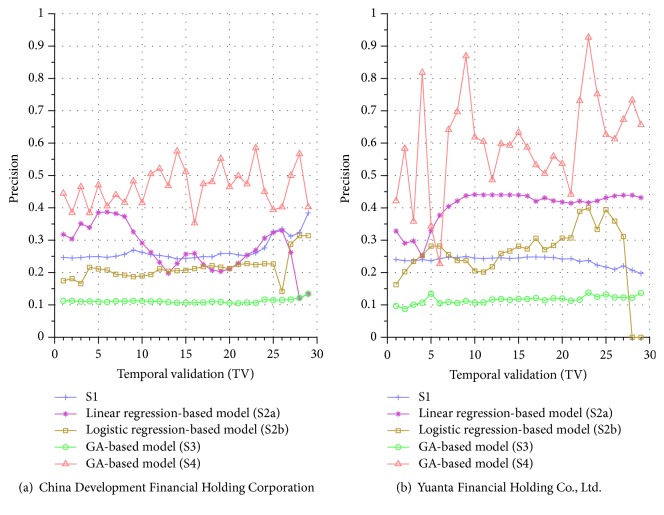
Precision comparison of the forecasting systems in the testing phase for 9/15/2015–11/3/2015.

**Figure 8:**
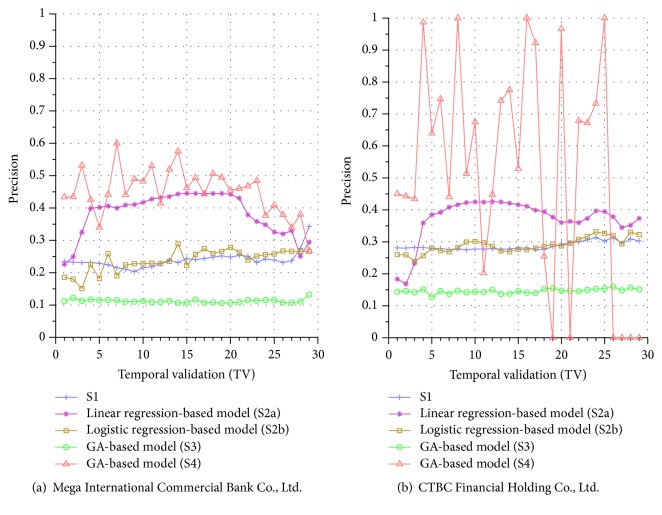
Precision comparison of the forecasting systems in the testing phase for 9/15/2015–11/03/2015.

Similar performance discrepancy of our proposed GA-based S4 model and other methods can be seen for the other stocks. For instance, Figure 4(b) shows the results for Siliconware Precision Industries Co., Ltd. Figures 5(a) and 5(b) show the results for Taiwan Semiconductor Manufacturing Company Limited and AU Optronics Corp., respectively. Figures 6(a) and 6(b) show the results for MediaTek Inc. and Cathay Financial Holding Co., Ltd. Figures 7(a) and 7(b) show the results for China Development Financial Holding Corporation and Yuanta Financial Holding Co., Ltd. Figures 8(a) and 8(b) show the results for Mega Financial Holding Company and CTBC Financial Holding Co., Ltd. As can be seen, the results in these ten stocks have shown that S4 outperforms S1, S2a, S2b, and S3 in most TV's, thereby indicating the effectiveness of our proposed S4 system.

**Figure 9:**
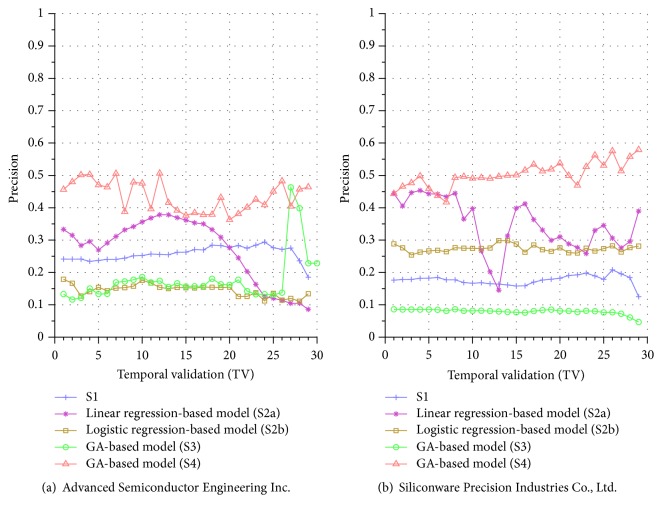
Precision comparison of the forecasting systems in the testing phase for 12/10/2015–1/21/2016.

**Figure 10:**
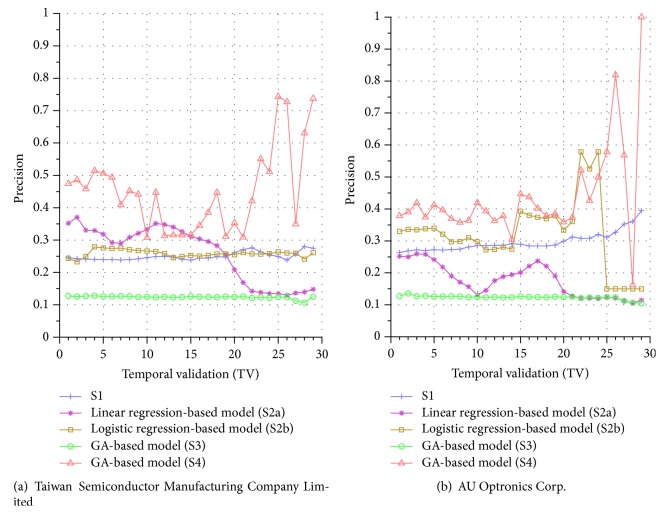
Precision comparison of the forecasting systems in the testing phase for 12/10/2015–1/21/2016.

**Figure 11:**
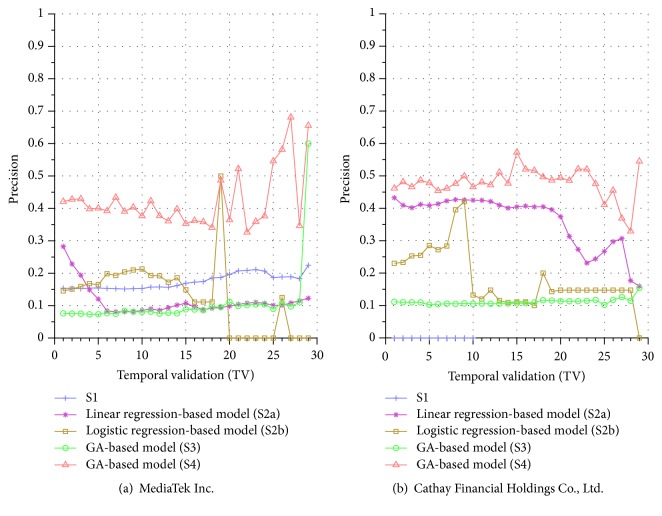
Precision comparison of the forecasting systems in the testing phase for 12/10/2015–1/21/2016.

**Figure 12:**
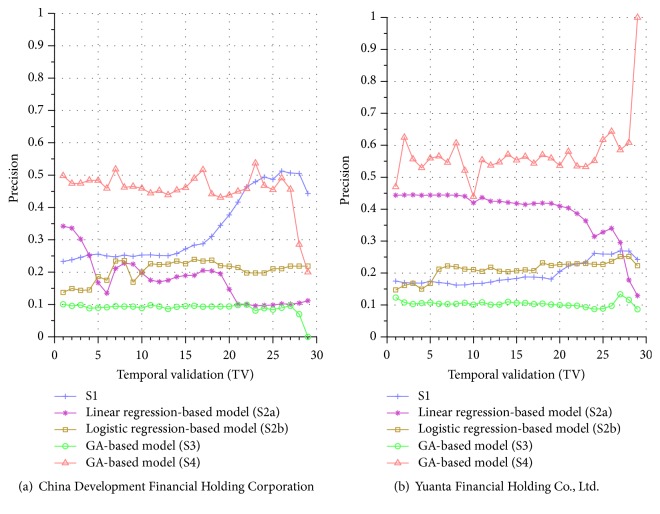
Precision comparison of the forecasting systems in the testing phase for 12/10/2015–1/21/2016.

**Figure 13:**
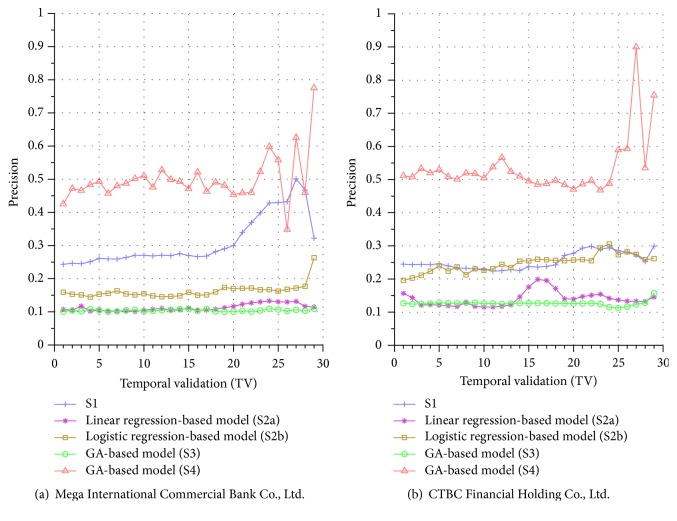
Precision comparison of the forecasting systems in the testing phase for 12/10/2015–1/21/2016.

The results just shown were obtained using the data from Sept./15/2015 to Nov./03/2015, during which time the broad stock market encountered favorable conditions and delivered positive gain as mentioned previously. In contrast, here we also display the results using the data from Dec./10/2015 to Jan./21/2016, during which time the market faced adverse situations and thus made loss. The results are shown in Figures 9(a) and 9(b) for Advanced Semiconductor Engineering Inc. and Siliconware Precision Industries Co., respectively. Figures 10(a) and 10(b) are for Taiwan Semiconductor Manufacturing Company Ltd. and AU Optronics Corp., respectively. Figures 11(a) and 11(b) are for MediaTek Inc. and Cathay Financial Holding Co., respectively. Figures 12(a) and 12(b) are for China Development Financial Holding Corporation and Yuanta Financial Holding Co., respectively. Figures 13(a) and 13(b) are for Mega Financial Holding Company and CTBC Financial Holding Co., respectively. As can be seen again, the results in these ten stocks have shown that S4 outperforms S1, S2a, S2b, and S3 in most TV's, thereby indicating the effectiveness of our proposed S4 system.

From these results, it appears that there is an upward trend of the precisions over training data size, for example, Figures 4(a), 4(b), 5(a), and 5(b) and others. However, there is also a downward trend of the precisions over training data size, for example, Figures 8(a) and 12(a), although the upward trend appears to be more frequent. For this situation, our conjecture is that, with more training data, the GA-based system S4 is more capable of learning to acquire better models for forecasting, and we intend to investigate this issue in the future.

Furthermore, in order to show the effectiveness of S4, Table 6 provides a summary that displays the mean and standard deviation computed for the precisions shown in each of Figures 4(a)–13(b). In this table, it can be seen that the higher value of the averaged precision for system S4 in each figure indicates this system outperforms the other systems, although many of the corresponding standard deviations are still relatively high. As we have already mentioned previously, this is again perhaps because the period of the testing phase in the final few TVs is not long enough for S4 to exhibit stable performance along temporal validations. Therefore, we intend to investigate this issue for the future work in order to achieve a more stable prediction performance for our proposed system S4.

**Figure 14:**
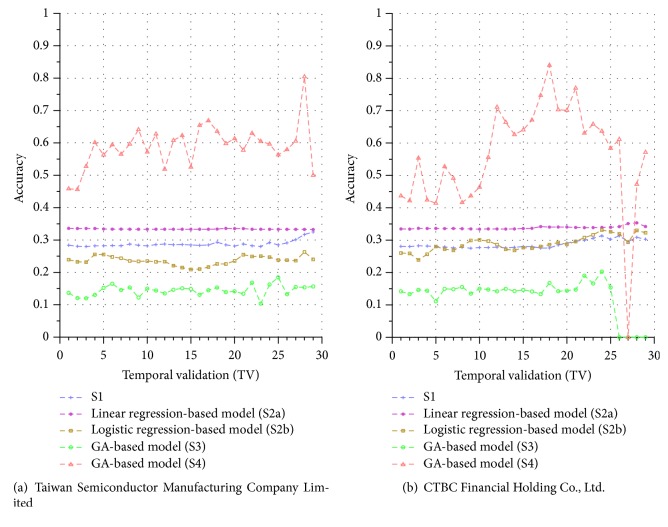
Accuracy comparison of the forecasting systems in the testing phase for 9/15/2015–11/3/2015.

**Figure 15:**
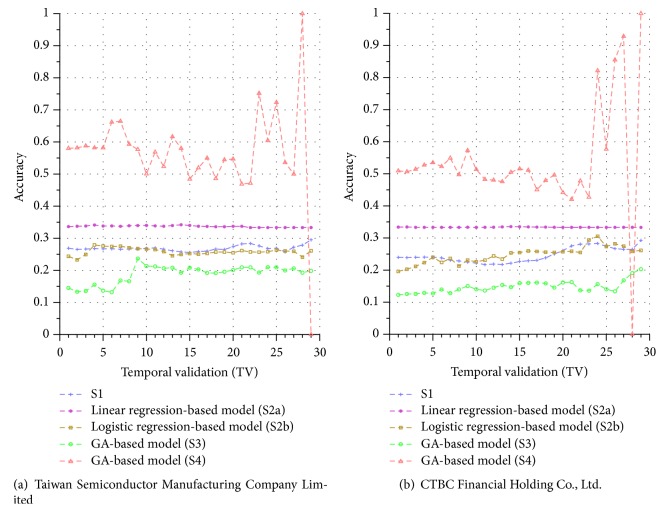
Accuracy comparison of the forecasting systems in the testing phase for 12/10/2015–1/21/2016.

All the results presented thus far were obtained using the precision metric by (7). For the accuracy metric, for illustrations, we display the results in Figures 14 and 15 using only two companies for the two periods of Sept./15/2015 to Nov./03/2015 and Dec./10/2015 to Jan./21/2016 (since the results for other companies are similar to those in these two figures, they are skipped here). As can be seen again, the results in Figures 14 and 15 have shown that S4 outperforms S1, S2a, S2b, and S3 in most TVs, thereby indicating that our proposed S4 system is more effective than others under the accuracy metric, as well.

## 4. Conclusions

In this paper, we presented a GA-based methodology for the research of high-speed trading. The particular domain of this study centers around the call auction market, which provides significant amount of price data of stocks at the microscopic level in financial markets nowadays. Through the optimization of parameters of the forecasting models, our experimental results showed that the proposed GA-based method is able to improve the accuracy of prediction for price movement on the microscopic level. In order to further examine the validity of our models, we conducted a statistical validation on the learned models and showed that our proposed models are effective in the dynamic environment of stock market, which is critical for practical investment where one expects the models constructed to gain profits in the future. With these results, we expect this GA-based method to advance the research in computational intelligence for financial applications and provide useful insights for high-speed trading.

In the future, since there exist several studies that use returns instead of prices for modeling, we thus propose to use returns for financial modeling as a potential line of research for high-speeding trading. Furthermore, since the results show that the precisions suffer high variance, we also intend to investigate this issue in more detail in order to reduce the variance in precisions, so that our proposed system can deliver more stable performance.

In addition, in this work, we have provided studies using the precision and accuracy metrics for performance evaluation. It may be worthwhile to conduct more detailed comparisons using these two metrics to investigate if both metrics have delicate discrepancy on the effectiveness of the models we proposed. Other alternatives are also possible; for instance, in the design for the fitness measure of a chromosome, we may somehow penalize its fitness by the variance of performance of the models. For other future work, we also intend to investigate the reason that led to the upward tendency of the precisions over increasing size of the training data. Our goal is to investigate if there exists a suitable size of training datasets for the GA-based method to acquire models that shall deliver more stable performance.

Furthermore, in this work, we employed simple GA with binary coding to search for the optimal parameters for the models. Indeed, there are various types of GA that could be employed for this study, as well. However, since the research of high-speed trading is a relatively new and unexplored area to computational intelligence, in this work, our major goal is thus to show, in principle, the GA is a useful tool for finding promising forecasting systems in the context of high-speed trading. In the future, we thus intend to employ more sophisticated versions of the GA in order to further improve the performance of our proposed systems.

## Figures and Tables

**Table 1:** Bid and ask quotes before matching (price in NT$ and volume in Lots).

Cumulative bid volume	Bid volume	Price	Ask volume	Cumulative ask volume
162	162	107.00	94	311
162		106.50	25	217
185	23	106.00	20	192
195	10	105.50	15	172
195		105.00	46	157
252	57	104.50	55	111
252		104.00	20	56
282	30	103.50	13	36
282		103.00	3	23
381	99	102.50		20
381		102.00		20
403	22	101.50		20
408	5	~~~		20
441	33	93.00	20	20

**Table 2:** Remaining unexecuted orders after matching.

Cumulative bid volume	Bid volume	Price	Ask volume	Cumulative ask volume
		107.00	94	119
		106.50	25	32
		106.00	7	7
10	10	105.50		
10		105.00		
67	57	104.50		
67		104.00		
97	30	103.50		
97		103.00		
196	99	102.50		
196		102.00		
218	22	101.50		
223	5	~~~		
256	33	93.00		

**Table 3:** Disclosure of information after matching.

Five best unexecuted quotes			
Bid price	Bid volume	Ask price	Ask volume
		107.00	94
		106.50	25
		106.00	7
105.50	10		
104.50	57		
103.50	30		
102.50	99		
101.50	22		

**Table 4:** Datasets of the 10 companies used in this study.

Ticker	Name (English)	Name (Chinese)	Market capitalization as of June/21/2016 ($NT billions)	Industry
2311	Advanced Semiconductor Engineering Inc.	日月光*半導體製*造*股份*有*限公司*	277.40	Semiconductor and electronics

2325	Siliconware Precision Industries Co., Ltd.	矽品*精密*工*業股份*有*限公司*	160.80	Semiconductor and electronics

2330	Taiwan Semiconductor Manufacturing Company Limited	台灣積體電路製造*股份*有*限公司*	4278.52	Semiconductor and electronics

2409	AU Optronics Corp.	友達光電*股份*有*限公司*	90.66	Semiconductor and electronics

2454	MediaTek Inc.	聯發科*技股份*有*限公司*	361.24	Semiconductor and electronics

2882	Cathay Financial Holding Co., Ltd.	國泰金*融控股股份*有*限公司*	474.89	Finance

2883	China Development Financial Holding Corporation	中華開發金*融控股股份*有*限公司*	117.61	Finance

2885	Yuanta Financial Holding Co., Ltd.	元大金*融控股股份*有*限公司*	123.92	Finance

2886	Mega Financial Holding Company	*兆豐*金*融控股股份*有*限公司*	333.20	Finance

2891	CTBC Financial Holding Co., Ltd.	中國*信託*商*業銀*行*股份*有*限公司*	298.81	Finance

**Table 5:** Averaged precision in training and testing in each TV.

TV	Averaged training precision					Averaged testing precision				
	S1	S2a	S2b	S3	S4	S1	S2a	S2b	S3	S4
1	0%	35.47%	26.05%	93.73%	97.25%	23.99%	31.23%	20.39%	12.37%	55.76%
2	25.94%	33.09%	26.30%	34.51%	100.00%	23.74%	33.08%	20.76%	12.12%	53.58%
3	29.66%	30.49%	26.17%	86.67%	98.43%	23.61%	29.27%	21.41%	12.28%	52.43%
4	28.58%	32.59%	24.80%	65.10%	99.61%	23.40%	28.10%	22.65%	12.12%	52.02%
5	28.61%	30.27%	23.38%	57.65%	100.00%	23.38%	29.52%	24.66%	11.95%	69.46%
6	27.73%	28.99%	19.79%	79.22%	100.00%	23.38%	30.57%	26.25%	11.99%	66.10%
7	26.84%	31.02%	19.85%	55.29%	100.00%	23.30%	31.47%	24.54%	11.81%	70.57%
8	26.56%	32.27%	20.32%	70.59%	99.22%	23.44%	32.81%	24.95%	11.92%	53.59%
9	25.69%	31.66%	19.67%	75.29%	100.00%	23.58%	33.81%	25.90%	12.26%	71.02%
10	25.07%	30.84%	18.93%	68.63%	98.82%	23.72%	34.93%	28.19%	12.23%	54.71%
11	24.62%	31.38%	19.56%	74.12%	98.82%	23.48%	34.12%	26.49%	12.09%	53.91%
12	24.95%	31.71%	19.85%	83.92%	100.00%	23.73%	34.03%	27.46%	12.23%	52.03%
13	24.43%	31.33%	20.74%	35.69%	100.00%	23.70%	34.79%	27.14%	12.49%	57.34%
14	24.41%	31.94%	21.14%	75.69%	100.00%	23.42%	35.49%	26.06%	12.21%	69.45%
15	24.67%	31.64%	21.79%	86.67%	98.43%	23.42%	36.47%	25.24%	12.19%	65.91%
16	24.59%	31.57%	21.46%	86.27%	99.22%	23.71%	37.99%	26.97%	12.27%	67.34%
17	24.26%	31.81%	21.68%	65.10%	99.61%	23.85%	40.03%	26.82%	12.21%	47.89%
18	24.13%	31.79%	21.79%	98.82%	100.00%	23.28%	41.43%	28.21%	12.38%	52.73%
19	24.46%	31.58%	22.43%	72.16%	81.18%	23.08%	43.05%	26.69%	11.96%	37.78%
20	24.50%	31.34%	22.94%	91.37%	99.61%	22.20%	43.84%	26.77%	11.61%	51.45%
21	24.82%	30.68%	23.54%	58.43%	99.22%	22.20%	44.10%	24.88%	11.80%	66.22%
22	24.72%	30.64%	23.38%	83.53%	100.00%	23.22%	43.76%	26.25%	11.88%	69.75%
23	24.27%	30.60%	24.90%	72.16%	99.22%	22.72%	43.86%	27.06%	11.44%	34.03%
24	24.38%	30.42%	24.97%	60.39%	99.61%	22.63%	43.75%	26.65%	11.37%	70.04%
25	24.34%	30.50%	24.58%	49.80%	99.61%	23.19%	43.36%	26.68%	11.65%	50.78%
26	24.17%	30.61%	24.82%	77.25%	100.00%	24.00%	42.31%	26.72%	12.35%	53.71%
27	24.01%	30.97%	24.98%	74.12%	99.61%	25.07%	41.36%	26.34%	13.06%	63.60%
28	23.90%	31.04%	25.16%	87.45%	99.22%	23.43%	43.10%	25.97%	10.37%	88.24%
29	24.05%	31.51%	24.93%	71.37%	98.43%	23.70%	42.09%	0.00%	13.48%	100.00%

**Table 6:** Averaged precisions and standard deviations for Figures 4(a)–13(b).

	Mean					Standard deviation				
	S1	S2a	S2b	S3	S4	S1	S2a	S2b	S3	S4
Figure 4(a)	0.2343	0.3737	0.2589	0.1207	0.6039	0.0055	0.0542	0.0606	0.0054	0.135
Figure 4(b)	0.2464	0.1611	0.2339	0.1267	0.5317	0.0061	0.026	0.0604	0.0041	0.0753
Figure 5(a)	0.2825	0.4191	0.2363	0.1375	0.6177	0.0063	0.0147	0.0137	0.027	0.1674
Figure 5(b)	0.2464	0.1611	0.1929	0.1267	0.5317	0.0061	0.026	0.0908	0.0041	0.0753
Figure 6(a)	0.1973	0.3394	0.2284	0.1042	0.5633	0.0121	0.085	0.0165	0.0123	0.0435
Figure 6(b)	0.2301	0.4189	0.2281	0.1196	0.4609	0.0108	0.0425	0.0203	0.0041	0.055
Figure 7(a)	0.2672	0.2747	0.214	0.1114	0.365	0.0346	0.0711	0.0375	0.0057	0.0605
Figure 7(b)	0.2366	0.4052	0.2589	0.1159	0.5988	0.0138	0.0526	0.0924	0.0116	0.1536
Figure 8(a)	0.2387	0.3822	0.2387	0.1124	0.451	0.0248	0.0663	0.0339	0.0055	0.0719
Figure 8(b)	0.2872	0.3717	0.2891	0.1461	0.526	0.0127	0.0665	0.0237	0.0069	0.348
Figure 9(a)	0.258	0.2714	0.1451	0.1768	0.4352	0.0226	0.0979	0.0181	0.0744	0.0464
Figure 9(b)	0.1764	0.3476	0.2737	0.0791	0.5032	0.0155	0.0803	0.0109	0.0081	0.0391
Figure 10(a)	0.25	0.26	0.2585	0.1236	0.4507	0.0123	0.0868	0.0108	0.0043	0.1299
Figure 10(b)	0.2966	0.1762	0.3215	0.1233	0.437	0.0304	0.0517	0.1119	0.006	0.1537
Figure 11(a)	0.1741	0.114	0.126	0.1054	0.4238	0.0225	0.0459	0.1092	0.0959	0.091
Figure 11(b)	0	0.3617	0.1808	0.1115	0.4782	0	0.0813	0.0899	0.0098	0.0475
Figure 12(a)	0.3336	0.1773	0.2049	0.0888	0.4533	0.105	0.07	0.0306	0.0182	0.0646
Figure 12(b)	0.1985	0.3915	0.2108	0.1036	0.5726	0.0371	0.0782	0.0274	0.0096	0.0921
Figure 13(a)	0.3109	0.1127	0.1624	0.1042	0.4986	0.0752	0.0105	0.0216	0.003	0.0732
Figure 13(b)	0.2537	0.1399	0.2472	0.1261	0.5351	0.0255	0.0226	0.026	0.0071	0.0885
